# Delayed Bleeding and Pelvic Haematoma after Low-Energy Osteoporotic Pubic Rami Fracture in a Warfarin Patient: An Unusual Cause of Abdominal Pain

**DOI:** 10.1155/2014/783268

**Published:** 2014-07-16

**Authors:** Andrea Sandri, Dario Regis, Nicola Bizzotto

**Affiliations:** Department of Orthopaedic and Trauma Surgery, Integrated University Hospital, 37126 Verona, Italy

## Abstract

*Introduction*. Acute abdominal pain may be the presenting symptom in a wide range of diseases in the elderly. Acute abdominal pain related to a delayed bleeding and pelvic haematoma after a low-energy pubic rami fracture is rare and can have important consequences; to the best of our knowledge, only one case has been previously described. *Case Report*. We present an unusual case of an 83-year-old woman taking warfarin for atrial fibrillation, admitted to the Emergency Department (ED) with acute abdominal pain and progressive anemia related to a delayed bleeding and pelvic haematoma 72 hours after a low-energy osteoporotic pubic rami fracture. Warfarin was withheld, anticoagulation was reversed by using fresh frozen plasma and vitamin K, and concentrated red blood cells were given. Haemoglobin level gradually returned to normal with a progressive resorption of the haematoma. *Conclusion*. Delayed bleeding and pelvic haematoma after osteoporotic pubic rami fracture should be considered in the differential diagnosis of acute abdominal pain in the elderly. This case indicates the need for hospital admission, careful haemodynamic monitoring, and early identification of bleeding in patients with “benign” osteoporotic pubic rami fracture, especially those receiving anticoagulants, to provide an adequate management and prevent severe complications.

## 1. Introduction

Osteoporotic pubic rami fractures are a common disease in older patients as a consequence of a moderate or minimal trauma [1–3]. These fractures are considered stable lesions and standard treatment includes bed rest, analgesics, and active mobilisation once the acute pain has resolved [3, 4].

Acute bleeding is one of the most common and severe complication in high-energy pelvic fractures [1, 5, 6]. However, the occurrence of these following low-energy pubic rami fractures in osteoporosis is rare but life-threatening [7–9].

This paper describes an unusual case of acute abdominal pain related to delayed bleeding and pelvic haematoma after a low-energy pubic rami fracture in an osteoporotic patient; to the best of our knowledge, only one case has been previously described [10]. A review of the literature, the diagnostic strategy, and treatment are discussed.

## 2. Case Report

**Figure 1:**
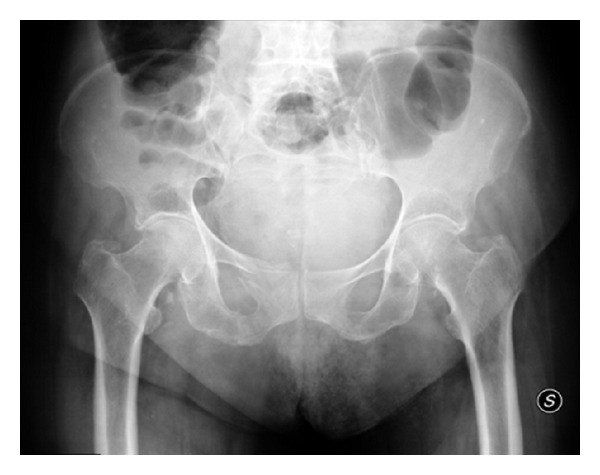
Anteroposterior pelvic radiograph revealing right superior and inferior pubic rami fractures.

An 83-year-old female was admitted to the Emergency Department (ED) with acute abdominal pain in the left lower quadrant. She had a medical history of osteoporosis and chronic atrial fibrillation, treated with alendronate and warfarin, respectively. Three days earlier, after a minor fall at home, she was treated at another local hospital with right hip pain and was discharged on the same day with a diagnosis of right osteoporotic pubic rami fracture (Figure 1) classified as type Ia according to the Rommens-Hofmann classification [11].

At admission to ED, blood pressure (BP) was 110/70 mmHg with a pulse rate of 120 beats per minute (BPM). Hemoglobin (Hb) was 11.3 gm/dL with an international normalized ratio (INR) of 3.25. White blood cell count, C-reactive protein, liver function, and amylase were within normal values.

**Figure 2:**
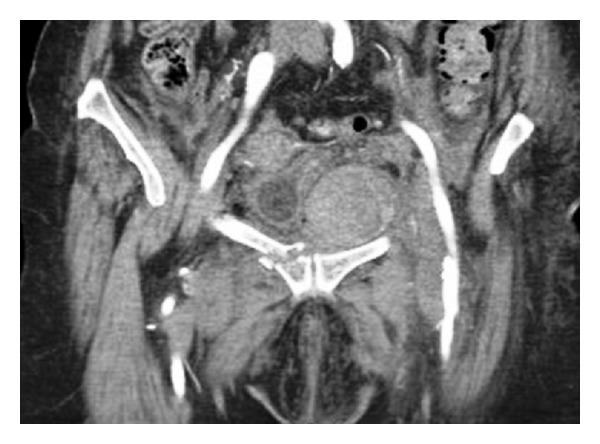
Coronal CT of the pelvis showing left haematoma neighboring the right fractured iliopubic ramus.

Clinical evaluation revealed a painful abdominal mass on the left lower abdominal quadrant with no signs of peritonism and hepatosplenomegaly. Rectal examination was normal. Abdominal ultrasonography was not useful in the evaluation of the retroperitoneum and the small pelvic cavity because of meteorism. Abdominopelvic computed tomography (CT) without contrast-enhanced revealed a left pelvic hematoma neighboring the fracture site (Figure 2).

**Figure 3:**
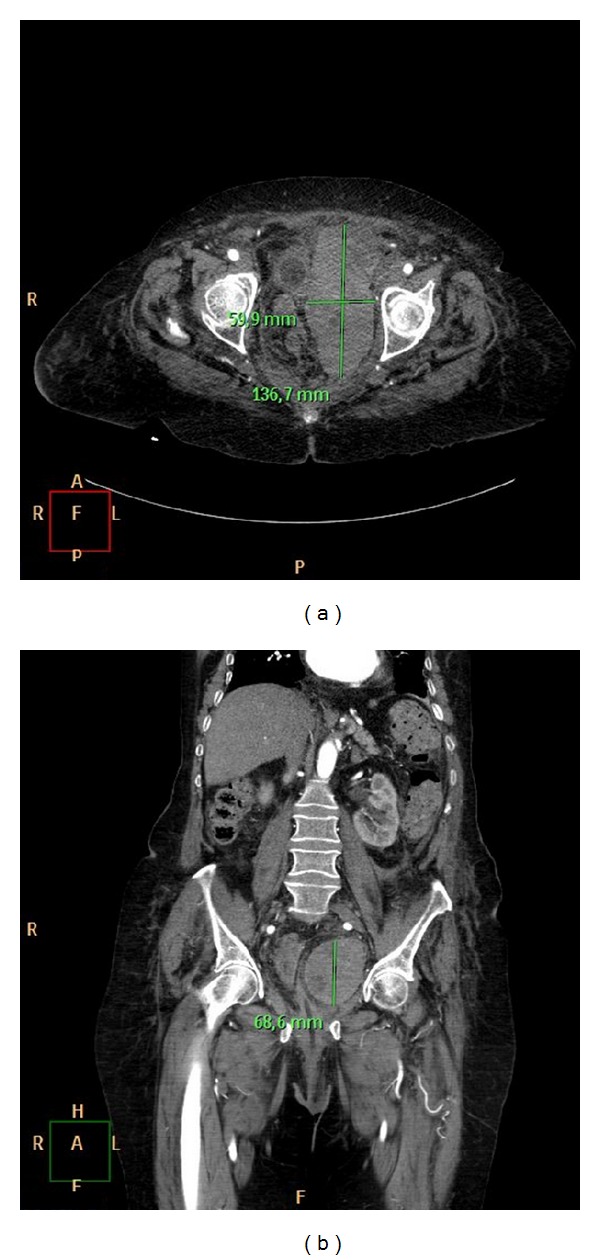
Axial (a) and coronal (b) CT of the pelvis demonstrating the size of pelvic haematoma.

Warfarin was withheld and anticoagulation was reversed by using 2 units of fresh frozen plasma (FFP) and 5 mg vitamin K. Her blood pressure remained stable on 120/80 mmHg with a pulse rate of 130 beats/min. However, four hours after admission Hb gradually dropped to 7.8 gm/dL. BP was 100/70 mmHg with 135 BPM, with a creatinine of 1,44 and INR of 2.07. A multidetector CT confirmed a pelvic haematoma with no active bleeding (Figure 3). She received 4 units of packed red blood cells as well as 2 units of FFP. Hb rose to 10.7 gm/dL, with an INR of 1.1. BP was 110/80 mmHg with a 80 BPM. Haemoglobin level and INR gradually returned to normal values and she remained haemodynamically stable with no evidence of recurrent pelvic haemorrhage throughout the hospital course. Abdomen ultrasonography was performed 5 and 10 days later, showing a progressive resorption of the haematoma. Twelve days after admission the patient was transferred to the cardiologic unit for a cardiac failure. The patient was discharged uneventfully for a cardiologic rehabilitation program on day 25. Four months later, the patient returned to her preinjury level of activity.

## 3. Discussion

Acute abdominal pain may be the presenting symptom in a wide range of diseases in the elderly and poses a difficult challenge for the emergency physician [12, 13]. Approximately 40% of the elderly with acute abdomen were misdiagnosed, and the overall mortality is approximately 10% [14]. Acute abdominal pain related to acute bleeding and pelvic haematoma after a low-energy pubic rami fracture has been rarely described [7, 15, 16]. Our patient was admitted to the hospital with abdominal pain associated with a delayed bleeding and pelvic haematoma 72 hours after trauma. Only Garrido-Gómez et al. [10] have described this delayed occurrence in a 70-year-old osteoporotic woman, who developed a painful abdominal mass and haemodynamic instability 72 hours following a left iliopubic rami fracture and nondisplaced right ischiopubic rami fracture after fall at home. An emergent multiphase contrast CT showed a large haematoma neighboring the fractured left iliopubic ramus and arterial bleeding. Selective angiography revealed an active haemorrhage from the distal portion of a small branch of the left obturator artery, which was treated successfully by embolization. In our patient, CT showed a large haematoma neighboring the pubic rami with no arterial bleeding; therefore, the conservative treatment with resuscitative measures, withheld anticoagulant therapy and reverse anticoagulation was adequate.

Acute bleeding after low-energy pubic rami fractures in osteoporosis is rare but life-threatening [7–9]. In the elderly, weakening of the supporting connective tissue of skeletal muscles renders vessels more prone to easy damage from minor trauma and atherosclerosis may limit the ability for injured vessels to develop vasospasm and spontaneously tamponade [17]. Several comorbidities and reduced cardiovascular reserve in the elderly limit the response to bleeding and a quick detection is critical to ultimate survival [2].

The options to treat bleeding in low-energy pubic rami fractures in the elderly depend on the source of bleeding. Bleeding may arise from injury of bone surfaces at the fracture site, muscles, pelvic veins, small arteries, or major vascular structures and requires prompt diagnostic and haemostatic procedures. Contrast-enhanced CT is useful to identify active bleeding and less invasive than conventional angiography, although the latter offers possibilities of embolization in protracted bleeding in unstable patients [18]. Haemorrhage from small-caliber veins, muscles, and broad cancellous bone surfaces is commonly self-limiting with a low flow, so the conservative management with appropriate resuscitative measures is usually sufficient to stop bleeding. Concurrent warfarin therapy may increase the risk of haemorrhage and concomitant analgesic therapy may elevate INR in patients receiving stable warfarin [19, 20]. Macdonald et al. [15] observed a massive retroperitoneal haematoma in a 71-year-old female patient receiving warfarin who sustained a superior pubic ramus fracture after a trivial fall at home. Bleeding arising from the pubic branch of the left inferior epigastric artery was treated by embolization, but the patient died from respiratory failure 48 hours after admission. Other authors have reported acute haemodynamic instability secondary to minimally displaced pubic rami in oral anticoagulant therapy treated by embolization of the injured vessels [15, 21]. In all the patients bleeding and haemodynamic instability occurred quickly, within 6 hours after the fracture.

In potentially life-threatening bleeding a rapid warfarin reversal is required to increase the ability to coagulate the bleeding vessels [20]. Fluid resuscitation must be continued during diagnostic and therapeutic procedures. A waiting period of 24 to 72 hours without clinical signs of bleeding is advised before restarting anticoagulation therapy [22]. The use of low-molecular-weight heparin may be necessary in patients with severe cardiovascular risk, but a careful monitoring for recurrent bleeding is recommended [23]. However, injuries of large veins or arteries produce a not self-limiting active bleeding and haemodynamic instability. The extravasation of contrast material on contrast-enhanced CT may be an indicator of potential arterial injury and angiographic embolization in unstable patients with protracted bleeding is required [5].

## 4. Conclusion

Delayed bleeding and pelvic haematoma after low-energy pubic rami fracture in osteoporotic patients should be considered in the differential diagnosis of acute abdominal pain in the elderly. This case indicates the need for hospital admission, careful haemodynamic monitoring, and early identification of bleeding in patients with “benign” osteoporotic pubic rami fracture, especially those receiving anticoagulants, to provide an adequate management and prevent severe complications.
